# Proteomic analysis of novel targets associated with TrkA-mediated tyrosine phosphorylation signaling pathways in SK-N-MC neuroblastoma cells

**DOI:** 10.1002/pmic.201200251

**Published:** 2013-01-23

**Authors:** Fang-Xiang Wu, Habtom Ressom, Michael J Dunn

**Affiliations:** 1 Department of Biochemistry and Institute of Health Sciences, Gyeongsang National University School of MedicineJinju, South Korea; 2 Department of Plastic and Reconstructive Surgery, School of Medicine, Kangwon National UniversityChuncheon, South Korea

**Keywords:** α-Enolase, α-Tubulin, Cell Biology, GW441756, hnRNP C1/C2, TrkA

## Abstract

Tropomyosin-related kinase A (TrkA) is a receptor-type protein tyrosine kinase and exploits pleiotypic roles via nerve growth factor (NGF)-dependent or NGF-independent mechanisms in various cell types. Here, we showed that the inhibition of TrkA activity by GW441756 resulted in the suppression of tyrosine phosphorylation of cellular proteins including extracellular signal-regulated protein kinase (ERK) and c-Jun N-terminal kinase (JNK). To find novel targets associated with TrkA-mediated tyrosine phosphorylation signaling pathways, we investigated GW441756 effects on TrkA-dependent targets in SK-N-MC neuroblastoma cells by proteomic analysis. The major TrkA-dependent protein spots controlled by GW441756 were determined by PDQuest image analysis, identified by MALDI-TOF MS and MALDI-TOF/TOF MS/MS, and verified by 2DE/Western blot analysis. Thus, we found that most of the identified protein spots were modified forms in a normal condition, and their modifications were regulated by TrkA activity. Especially, our results demonstrated that the modifications of α-tubulin and heterogeneous nuclear ribonucleoproteins C1/C2 (hnRNP C1/C2) were significantly upregulated by TrkA, whereas α-enolase modification was downregulated by TrkA, and it was suppressed by GW441756, indicating that TrkA activity is required for their modifications. Taken together, we suggest here that the major novel TrkA-dependent targets such as α-tubulin, hnRNP C1/C2, and α-enolase could play an essential role in TrkA-mediated tyrosine phosphorylation signaling pathways via regulation of their posttranslational modifications.

## 1 Introduction

In response to nerve growth factor (NGF), tropomyosin-related kinase A (TrkA) is dimerized and phosphorylated on several tyrosine residues in the catalytic domain (Y-670, Y-674, and Y-675) and noncatalytic intracellular domain (Y-490 and Y-785) 1,2. It has been known that NGF-induced autophosphorylation of Y-670, Y-674, and Y-675 is essential for TrkA activity and causes trans-phosphorylation of TrkA at Y-490 and Y-785, resulting in the activation of NGF-mediated TrkA signaling 3. The activated TrkA recruits and phosphorylates the adaptor protein Shc via Y-490 phosphorylation, and thus the phosphorylated Shc associates with the adaptor protein Grb2 and the Ras exchange factor Sos, leading to the activation of Ras signaling 4. NGF-induced Y-490 phosphorylation of TrkA is upregulated by constitutively active Src in SK-N-MC cells, causing enhancement of extracellular signal-regulated protein kinase (ERK) signaling 5. However, interaction of TrkA with SHP-1, a phosphotyrosine phosphatase, via Y-490 phosphorylation induces dephosphorylation at Y-674 and Y675, leading to the inhibition of NGF-mediated cellular processes including phosphorylation of ERK, phospholipase C-γ (PLC-γ), and Akt 6. These results suggest that the cellular effects of TrkA through Y-490 phosphorylation are dependent on the downstream signaling pathways. Y-785 phosphorylation is required for the interaction of TrkA with PLC-γ, resulting in the activation of Ras signaling followed by NGF-induced apoptosis in medulloblastoma cells 7. Mutation of TrkA at Y-785 (Y785F) protects from the apoptosis induced by either c-Myc or UV treatment, whereas mutation of TrkA at Y-490 (Y490F) inhibits c-Myc-induced apoptosis but not UV-, showing a differential apoptotic signaling pathways on the tyrosine phosphorylation resides of TrkA 8. Paradoxically, it has been well known that TrkA plays an important role in the signal transduction of cell survival in response to NGF 1,2,9,10. Until now, differential mechanisms of cell survival and death by TrkA are still unclear. Nonetheless, Y-490 phosphorylation, at least, seems to be essential for both TrkA-induced cell survival and death.

Intriguingly, NGF-independent activation of TrkA can be induced by various stimuli including amyloid-beta 11,12, proteosome inhibitors such as MG-132 and lactacystin 13,14, and dorsomorphin, a selective inhibitor of bone morphogenetic protein signaling 15. Inhibition of proteasome activity causes intracellular protein aggregation, which has been quite observed in a neurodegenerative disorder such as Alzheimer's disease, Parkinson's disease and amyotrophic lateral sclerosis 16,17. The microtubule-associated protein tau is one of the major aggregating proteins, and its aggregation is predominantly associated with phosphorylation by the protein kinases such as tau-tubulin kinase 1, glycogen synthase kinase 3β, and cyclin-dependent protein kinase CDK5/p25 complexes 18,19. The phosphorylation downregulates tau interactions with phosphatidylinositol 3-kinase, PLC-γ, Grb2, and Src family kinases, resulting in the disruption of the intracellular networks of tau 20. Moreover, Tau is associated with Aβ-induced axonal transport defects 21, and Aβ regulates posttranslational modification of tau and tubulin followed by microtubule disruption 22.

We have previously showed that TrkA overexpression predominantly chose cell death rather than cell survival in both neuronal SK-N-MC cells and non-neuronal U2OS cells in an NGF-independent mechanism 23. To better understand TrkA-mediated intracellular pathways, we have investigated novel TrkA-dependent targets associated with tyrosine phosphorylation signaling pathways using the inhibition effect of TrkA activity by GW441756 in SK-N-MC neuroblastoma cells by proteomic analysis. We show here that α- and β-tubulin, heterogeneous nuclear ribonucleoproteins C1/C2 (hnRNP C1/C2), peroxiredoxin-6, and α-enolase are novel TrkA-dependent targets and could play an important role in TrkA-mediated tyrosine phosphorylation signaling pathways via regulation of their posttranslational modifications.

## 2 Materials and methods

### 2.1 Reagents

DMEM, tetracycline-screened FBS, and penicillin/streptomycin were from Gibco-BRL. Tetracycline (Tet) was from Duchefa. Blasticidine and zeocin were from Invitrogen. Aqueous mounting media was from Biomeda. Twenty percent formaldehyde was from Tousimis. Super signal west pico stable peroxide solution was from Pierce. Protran nitrocellulose (NC) membrane (BA83) was from Whatman. GW441756, iodoacetamide, trichloroacetic acid, Bradford reagent, Commassie Brilliant Blue G, octyl β-D-1-thioglucopyranoside, and α-cyano-4-hydroxycinnamic acid (CHCA) were from Sigma-Aldrich. Forty percent acrylamide/bis-acrylamide 37.5:1 solution, urea, thiourea, CHAPS, Tris, Tween-20, ammonium sulfate, and DTT were from Amresco. Bio-Lyte 3/10 ampholyte, IPG strip (pH 4–7, 7 cm), IPG strip (pH 4–7, 17 cm), mineral oil, and protean plus overlay agarose were from Bio-Rad. IPG buffer (pH 4–7) was from Amersham Biosciences. Bromophenol blue was from Pharmacia Biotech. Recombinant DNase I (RNase-free) was from Takara. Protease inhibitor cocktail set I was from Calbiochem. Ortho-phosphoric acid (85%) was from Merck. Sequencing grade-modified trypsin was from Promega. Antibodies used in the study were: TrkA (763, Santa Cruz), phospho-TrkA (E-6, Santa Cruz), phospho-Tyr (Transduction Laboratories), phospho-Y204-ERK (Bioworld Technology), phospho-T183/Y185-JNK (Bioworld Technology), β-actin (Sigma-Aldrich), α-tubulin (B-5–1-2, Sigma-Aldrich), acetylated α-tubulin (6–11B-1, Santa Cruz), β-tubulin (H-235, Santa Cruz), hnRNP C1/C2 (4F4, Santa Cruz), α-enolase (L-27, Santa Cruz), secondary goat anti-rabbit and anti-mouse HRP conjugates (Bio-Rad), and anti-rabbit TRITC conjugate (Sigma-Aldrich).

### 2.2 Cell culture

We have previously established TrkA-inducible stable cells, SK-N-MC-TrkA #9 and #21, which can induce TrkA overexpression in the presence of tetracycline but not in the absence 23. The cells were maintained with medium A [DMEM, tetracycline-screened 10% FBS, 1% penicillin/streptomycin] containing 1.25 μg/mL blasticidine and 25 μg/mL zeocin in a humidified 5% CO_2_ incubator at 37°C. TrkA overexpression in SK-N-MC-TrkA cells was induced by adding of 2 μg/mL tetracycline in the medium A for the indicated times.

### 2.3 Western blot analysis

Whole cells were extracted with SDS sample buffer and boiled for 5–10 min at 95°C. Proteins were separated on SDS polyacrylamide gel and transferred to NC membrane. The blot was blocked for 1 h at room temperature in blocking buffer (3% skim milk/0.1% Tween-20/PBS) and then incubated with primary antibody at 4°C overnight. The blot was washed with PBST (0.1% Tween-20/PBS) for 15 min three times and incubated with HRP-conjugated secondary antibody in blocking buffer for 2 h. After washing with PBST, the blot was analyzed by the super signal ECL detection system.

### 2.4 Confocal immunofluorescence microscopy

SK-N-MC-TrkA cells (#9 and #21) were cultured in the absence (–Tet) or presence (+Tet) of tetracycline on 6-well dish with a cover slide for 20 h. The cells were washed with PBS, fixed with 3% formaldehyde/PBS for 45 min, permeated with 0.5% Triton X-100/PBS for 3–5 min, blocked with 1% BSA/PBS for 1 h, and then incubated with anti-TrkA antibody for 2 h. After washing with PBS, the cells were incubated with anti-rabbit TRITC conjugated antibody for 1 h, washed with PBS and mounted with aqueous mounting media. Cell morphology and intracellular localization of protein were analyzed by confocal microscopy (Olympus FV-500).

### 2.5 Sample preparation for 2DE

Whole cells were washed with PBS, suspended in 2DE lysis buffer (8 M urea, 4% CHAPS, 40 mM Tris, 100 mM DTT, 0.5% Bio-Lyte 3/10 ampholyte, 1X protease inhibitor cocktail) using 22-gauge syringe needle, and then incubated on ice for 30 min. The cell lysates were treated with 20 U/mL of DNase I on ice for 1 h and precipitated with trichloroacetic acid (15–20%). After washing with acetone, the pellet was dissolved in rehydration solution (7 M urea, 2 M thiourea, 2% CHAPS, 100 mM DTT, 0.0002% bromophenol blue) followed by protein quantification using Bradford reagent.

### 2.6 2DE and Commassie Blue stain

Protein samples (500 μg each) were added with 0.5% IPG buffer (pH 4–7) and rehydrated with 17 cm IPG strip (pH 4–7) for 14 h at 20°C in the isoelectric focusing (IEF) cell followed by 1D isoelectric focusing in a maximum current of 50 μA/IPG strip; at 250 V for 15 min, at 10 000 V for 3 h, and then at 10 000 V for 65 000 V-h. The strips were equilibrated in buffer I (50 mM Tris (pH 8.8), 6 M urea, 30% glycerol, 2% SDS, 10 mg/mL DTT) for 15 min and in buffer II (50 mM Tris (pH 8.8), 6 M urea, 30% glycerol, 2% SDS, 25 mg/mL iodoacetamide) for 15 min. The 2D electrophoresis of the strips was performed on 8.5–14% sucrose gradient polyacrylamide gel prepared using the light 8.5% SDS-PAGE solution and the heavy 14% SDS-PAGE solution containing 15% sucrose; at 20 mA for 1 h and subsequently maintain with 30 mA during electrophoresis. The gels were fixed in fixing solution (40% methanol, 5% ortho-phosphoric acid) at room temperature for 3 h and stained with Commassie Blue staining solution (17% ammonium sulfate, 3% phosphoric acid, 34% methanol, 0.1% Commassie Brilliant Blue G) for 24 h. After destaining with H_2_O, gel images were obtained using a UMAX scanner (PowerLook 2100XL).

### 2.7 Image analysis and statistical significance

Quantitative analysis of Commassie Blue stained images was carried out using PDQuest software (Bio-Rad) according to the manufacturer's instructions. Quantity of each spot was normalized by total valid spot intensity. Protein spots associated with TrkA-mediated tyrosine phosphorylation signaling pathways were determined by the inhibitory effects of GW441756 on upregulated or downregulated TrkA-dependent targets in SK-N-MC cells. The statistical significance of image analysis was determined by the Student's *t*-test (statistical level of *p* < 0.05 is significant).

### 2.8 In-gel tryptic digestion

The target spots stained with Commassie Blue were excised and prepared for MALDI-TOF MS analysis in a manner similar to that previously described 24 with a slight modification. Briefly, excised spots were destained with 50% acetonitrile solution, reduced in 10 mM DTT/100 mM NH_4_HCO_3_ solution for 45 min at 56°C, and alkylated in 55 mM iodoacetamide/100 mM NH_4_HCO_3_ solution for 30 min in the dark. The gel pieces were incubated for 5 min in 100 mM NH_4_HCO_3_ and then for 15 min after adding an equal volume of acetonitrile. After vacuum dry, the gel pieces were treated with 20 μL of trypsin digestion solution (50 mM NH_4_HCO_3_, 0.01% octyl β-D-1-thioglucopyranoside, 12.5 μg/mL trypsin) for 13–15 h at 37°C. The tryptic-digested peptides (2 μL) were mixed with 2 μL of saturated matrix solution (15 mg CHCA, 15 mg NC membrane, 75% acetone, 25% 2-propanol) and 1 μL of mixed calibrants (bradykinin (0.28 pmole/μL) and neurotensin (0.15 pmole/μL)). The mixed tryptic peptides (1.5 μL) were immediately spotted onto stainless steel MALDI-TOF sample target plate.

### 2.9 MALDI-TOF MS and database searching

Mass measurement of tryptic peptides was performed with MALDI-TOF MS (Voyager-DE-STR; Applied Biosystems), and mass spectra were acquired for the mass range of 800–3500 Da and calibrated with Bradikinin *m/z* (904.4681) and Neurotensin *m/z* (1672.9175) as standard peaks. The proteins were identified by peptide mass fingerprinting (PMF) on the basis of the Swiss-Prot database using the search program MASCOT (http://www.matrixscience.com), allowing peptide mass tolerance of 1.2 Da and one missed cleavage. Significance of the searched data was judged from the scores more than 56 (*p* < 0.05) and at least seven matched peptide masses.

### 2.10 MALDI-TOF/TOF MS/MS and database searching

Protein spots were excised from Commassie Blue stained 2DE gels and performed in-gel digestion using modified porcine trypsin as described 25. Peptide mass spectra were obtained by MALDI-TOF/TOF MS/MS (ABI 4800 plus; Applied Biosystems), and internal calibration was accomplished by analysis of autolytic trypsin cleavage products. Search for protein identity was carried out using the NCBInr database by ProteinPilot v.3.0 (with MASCOT as the database search engine) with peptide and fragment ion mass tolerance of 50 ppm 26. The other parameters for searching were one missed trypsin cleavage, carbamidomethylation of cysteine, oxidation of methionine, and monoisotopic. Significance of the identified proteins was based on the number of matching peptide masses and comparison of experimental and theoretical properties of the proteins, in addition to database searched protein scores; greater than 84 were considered as a statistically significant (*p* < 0.05).

### 2.11 Confirmation of the identified proteins by 2DE/Western blot analysis

Protein samples (10 μg each) for 7 cm IPG strip (pH 4–7) were added with 0.5% IPG buffer (pH 4–7) and rehydrated at 20°C for 14 h in the isoelectric focusing cell followed by 1D isoelectric focusing in a maximum current of 50 μA/IPG strip; at 250 V for 15 min, at 4000 V for 2 h, and then at 4000 V for 30 000 V-h. The strips were equilibrated in buffer I for 15 min and subsequently in buffer II for 15 min, and then performed by 10% SDS-PAGE. The protein spots were transferred to NC membrane and analyzed by Western blot. Protein samples (50 μg each) for 17 cm IPG strip (pH 4–7) were separated by 2DE using 8.5–14% sucrose gradient polyacrylamide gels as described above. The areas of the identified protein spots were excised and transferred to NC membrane followed by Western blot analysis.

## 3 Results

### 3.1 TrkA overexpression causes morphological change of SK-N-MC neuroblastoma cells

**Figure 1:**
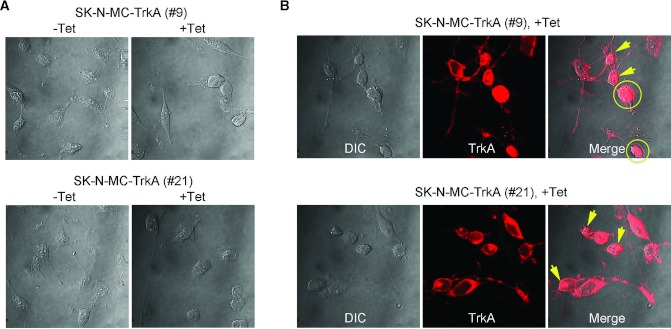
Morphological change of SK-N-MC neuroblastoma cells by TrkA overexpression. SK-N-MC-TrkA cells (stable clones #9 and #21) were cultured in the absence (–Tet) or presence (+Tet) of tetracycline for 20 h. The cells were fixed and then stained with TRITC-labeled red against anti-TrkA antibody. Differential interference contrast (DIC) image and the intracellular localization of ectopic TrkA were analyzed by confocal microscopy. Merge panel shows combined image of DIC and TrkA.

It has been reported that ectopic expression of TrkA induces apoptosis of neuroblastoma cells in a p53-dependent mechanism 27. We have previously showed that TrkA overexpression resulted in a significant cell death and morphological change of U2OS cells to a neuron-like phenotype 28. Here, we investigated the effects of TrkA overexpression on the morphology of SK-N-MC neuroblastoma cells. As shown in Fig. 1A, TrkA-inducible stable cell lines, #9 and #21, were changed to a considerable long cell body or rounded phenotype in the presence of TrkA expression (+Tet panel) but not in the absence (–Tet panel). TrkA expression was predominantly located in the cytoplasm with a large aggresome around nucleus in both #9 and #21 cells (see yellow-colored arrows in Fig. 1B). Unexpectedly, TrkA expression was also detected in the nucleus of certain cells (see yellow-colored circles in Fig. 1B), suggesting a nuclear trafficking of TrkA by an unknown mechanism.

### 3.2 TrkA-mediated cellular processes are suppressed by TrkA inhibitor GW441756

**Figure 2:**
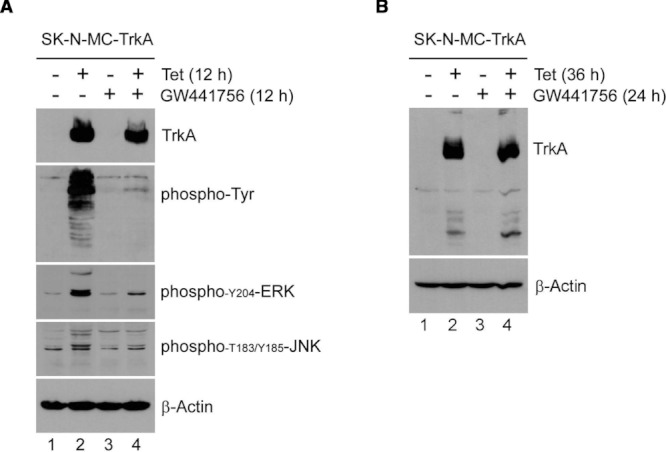
GW441756 effects on the cellular processes by TrkA overexpression. (A) SK-N-MC-TrkA cells (#9) were untreated or treated with 2 μM of GW441756 for 12 h in the absence or presence of tetracycline. (B) SK-N-MC-TrkA cells (#9) were cultured in the absence or presence of tetracycline for 12 h and then untreated or treated with 2 μM of GW441756 for 24 h, leading to 36 h Tet-On induction. Whole cell extracts were analyzed by Western blot using antibodies against TrkA, phospho-Tyr, phospho-Y204-ERK, phospho-T183/Y185-JNK, and β-actin.

We have previously shown that TrkA overexpression induced various cellular processes such as ERK and JNK phosphorylation, PARP cleavage, and γH2AX production in U2OS osteosarcoma cells 28,29. In this report, we show that TrkA overexpression causes tyrosine phosphorylation of cellular proteins including ERK and JNK in SK-N-MC neuroblastoma cells, and these cellular processes are blocked by TrkA inhibitor GW441756 (Fig. 2A). It has been reported that NGF-induced neurite outgrowth was significantly suppressed by the inhibition of ERK and JNK activation in PC12 cells 30. These results suggest that the activated ERK and JNK could be involved in the morphological change of SK-N-MC neuroblastoma cells by TrkA overexpression (Fig. 1). Notably, TrkA expression had no significant change by GW441756 at 12 and 36 h after tetracycline treatment (Fig. 2A and B).

### 3.3 2DE and image analysis of GW441756 effects on TrkA-dependent targets

**Figure 3:**
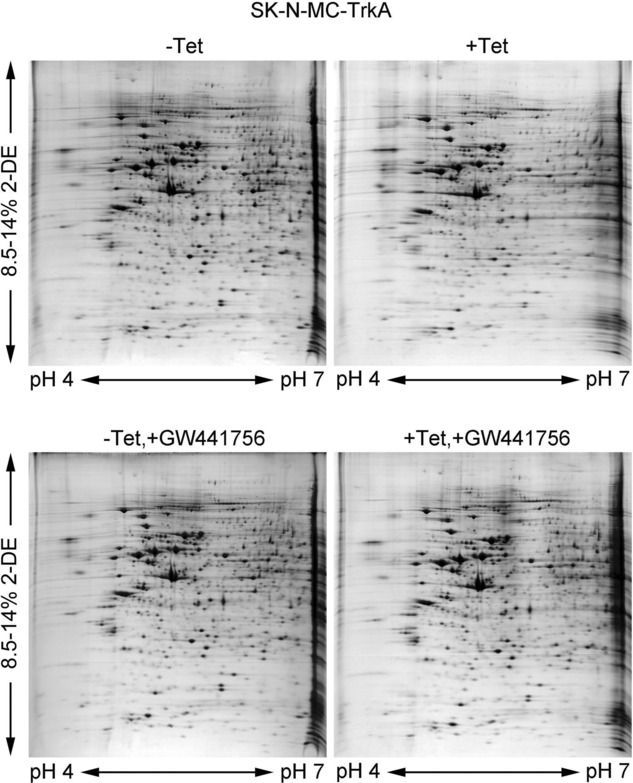
Representative 2DE images of GW441756 effects on TrkA-dependent targets. SK-N-MC-TrkA cells (#9) were cultured in the absence or presence of tetracycline for 12 h and then untreated or treated with 2 μM of GW441756 for 24 h. The cells were analyzed by 2DE and Commassie Blue stain.

To find novel targets associated with TrkA-mediated tyrosine phosphorylation signaling pathways, we investigated the effects of GW441756 at 36 h after tetracycline treatment in SK-N-MC-TrkA cells by 2DE/Commassie Blue stain analysis. This study was carried out five times in an independent experiment, and representative 2DE images have shown in Fig. 3. PDQuest image analysis was performed with two sets of well-separated 2DE images, and then ten protein spots were determined as major TrkA-dependent targets influenced by GW441756.

### 3.4 Identification of GW441756-controlled TrkA-dependent protein spots

We performed MALDI-TOF MS to identify the ten TrkA-dependent protein spots regulated by GW441756. The peptide mass spectra treated with trypsin were clearly acquired for the mass range of 800–3500 Da (data not shown) and analyzed by peptide mass fingerprinting using the search program MASCOT (http://www.matrixscience.com). As shown in Table 1, the ten protein spots were identified as pleckstrin homology domain-containing family O member 2 (SSP 1122), β-tubulin (SSP 2424), hnRNP C1/C2 (SSP 3209), α-tubulin (SSP 4506, 4532, 5416), α-enolase (SSP 7420), peroxiredoxin-6 (SSP 8101), uncharacterized protein C12orf56 (SSP 8105), and proteosome subunit alpha type-6 (SSP 8106) with a statistical significance (*p* < 0.05).

**Table 1:** Protein identification of GW441756-controlled TrkA-dependent targets by MALDI-TOF MS analysis

Spot no.	Protein identification by MALDI-TOF MS	Tryptic fragment coverage/matches	MASCOT probability score/expect (*p*)	UniProtKB/Swiss -Prot accession	Protein mass (Da)	p*I*
1122	Pleckstrin homology domain-containing family O member 2	15%/7 fragment	78/0.00034	Q8TD55	53317	5.34
2424	Tubulin beta chain (β-tubulin)	19%/7 fragment	81/0.00015	P07437	49639	4.78
3209	Heterogeneous nuclear ribonucleoproteins C1/C2 (hnRNP C1/C2)	19%/7 fragment	70/0.002	P07910	33650	4.95
4506	Tubulin alpha-1C chain (α-tubulin)	25%/8 fragment	97/4.2e-06	Q9BQE3	49863	4.96
4506	Tubulin alpha-1B chain (α-tubulin)	25%/8 fragment	97/4.4e-06	P68363	50120	4.94
4532	Tubulin alpha-1B chain (α-tubulin)	29%/9 fragment	111/1.6e-07	P68363	50120	4.94
5416	Tubulin alpha-1B chain (α-tubulin)	23%/8 fragment	97/3.9e-06	P68363	50120	4.94
7420	Alpha-enolase (α-enolase)	19%/7 fragment	78/0.00034	P06733	47139	7.01
8101	Peroxiredoxin-6	24%/7 fragment	90/2.3e-05	P30041	25019	6.00
8105	Uncharacterized protein C12orf56	13%/8 fragment	70/0.0019	Q8IXR9	71288	9.26
8106	Proteasome subunit alpha type-6	35%/8 fragment	108/3.2e-07	P60900	27382	6.34

Peptide profiles of the ten protein spots were analyzed by MALDI-TOF MS. The search program MASCOT (http://www.matrixscience.com) was used for identification using peptide mass fingerprinting.

To confirm the identification results by MALDI-TOF MS analysis, the ten protein spots were reanalyzed by MALDI-TOF/TOF MS/MS in an independent experiment. Thus, the identification results of seven protein spots have shown in Table 2 with a statistical significance (*p* < 0.05), and they were well matched with those analyzed by MALDI-TOF MS. However, SSP 8106 was identified as glutathione S-transferase by MALDI-TOF/TOF MS/MS analysis (data not shown) but proteosome subunit alpha type-6 by MALDI-TOF MS analysis. Judging from theoretical and experimental properties of molecular weight and p*I* SSP 8106 seems to be, at present, potentially proteosome subunit alpha type-6. Unfortunately, the identification results of SSP 1122 and 8105 by MALD-TOF/TOF MS/MS analysis were not statistically significant (data not shown).

**Table 2:** Protein identification of GW441756-controlled TrkA-dependent targets by MALDI-TOF/TOF MS/MS analysis

Spot no.	Protein identification by MALDI-TOF/TOF MS/MS	Tryptic fragment coverage/matches	MASCOT probability score/expect (*p*)	UniProtKB/ EMBL accession	Protein mass (Da)	p*I*
2424	Tubulin beta polypeptide (β-tubulin)	53%/35 fragment	407/3e-034	Q5JP53	48135	4.70
3209	Heterogeneous nuclear ribonucleoproteins C1/C2 (hnRNP C1/C2)	16%/6 fragment	91/0.011	G3V576	25298	9.82
4506	cDNA FLJ32131 fis (α-tubulin)	40%/24 fragment	419/1.9e-035	B3KPS3	46725	4.99
4532	cDNA FLJ32131 fis (α-tubulin)	38%/20 fragment	314/5.9e-025	B3KPS3	46725	4.99
5416	cDNA FLJ32131 fis (α-tubulin)	38%/20 fragment	267/3e-020	B3KPS3	46725	4.99
7420	Alpha-enolase (α-enolase)	38%/25 fragment	237/3e-017	G2HEB0	47516	6.63
8101	Peroxiredoxin-6	36%/9 fragment	183/7.5e-012	Q5R7E0	25119	6.00

The protein spots identified by MALDI-TOF MS were reanalyzed by MALDI-TOF/TOF MS/MS in an independent experiment as described in Section 2.

### 3.5 Quantitative inhibition effects of GW441756 on TrkA-dependent targets

**Figure 4:**
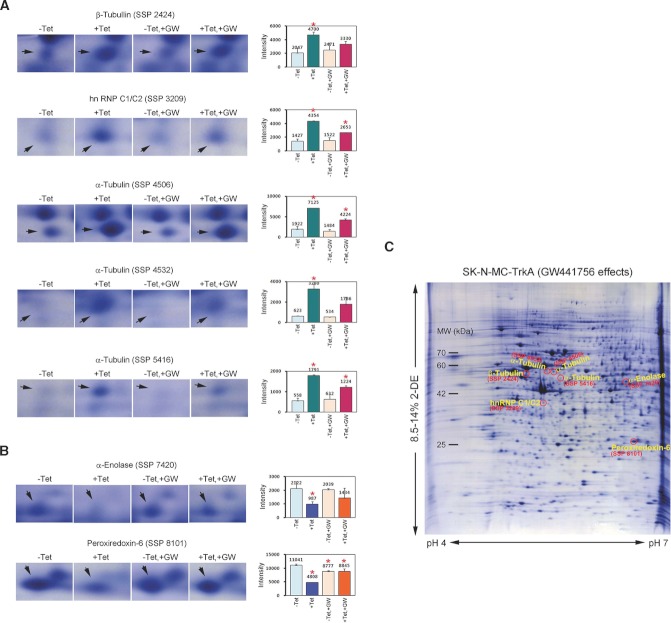
Quantitative analysis of GW441756 inhibitory effects on TrkA-dependent protein spots. PDQuest image analysis of GW441756 effects on TrkA-dependent protein spots was performed with two sets of Commassie Blue stained 2DE images. Seven protein spots, which had same identification results in MALDI-TOF MS and MALDI-TOF/TOF MS/MS analysis, were more than twofold upregulated (A) or downregulated (B) by TrkA overexpression, and it was suppressed by GW441756 (compare protein spots indicated by arrows in left four panels). Relative intensities of the protein spots determined by PDQuest software have shown in right panels. Each bar represents the mean ± SD for two independent experiments, and data significance was evaluated with a Student's *t*-test, *p < 0.05. (C) 2DE profile of GW441756-controlled TrkA-dependent protein spots that had matched identification results in MALDI-TOF MS and MALDI-TOF/TOF MS/MS analysis.

Protein expressions of the identified β-tubulin (SSP 2424), hnRNP C1/C2 (SSP 3209), and α-tubulin (SSP 4506, 4532, 5416) spots were more than twofold upregulated by TrkA with a statistical significance (*p < 0.05), and each TrkA-dependent upregulation was significantly inhibited by GW441756 (Fig. 4A). In contrast, protein expressions of the identified α-enolase (SSP 7420) and peroxiredoxin-6 (SSP 8101) spots were more than twofold downregulated by TrkA with a statistical significance (*p < 0.05), and it was inhibited by GW441756 (Fig. 4B). 2DE profiles of the seven GW441756-controlled TrkA-dependent protein spots have shown in Fig. 4C. Notably, the identified three spots (SSP 4506, 4532, 5416) were determined as α-tubulin with a different mobility, suggesting certain posttranslational modifications of α-tubulin as described previously 31,32.

**Figure 5:**
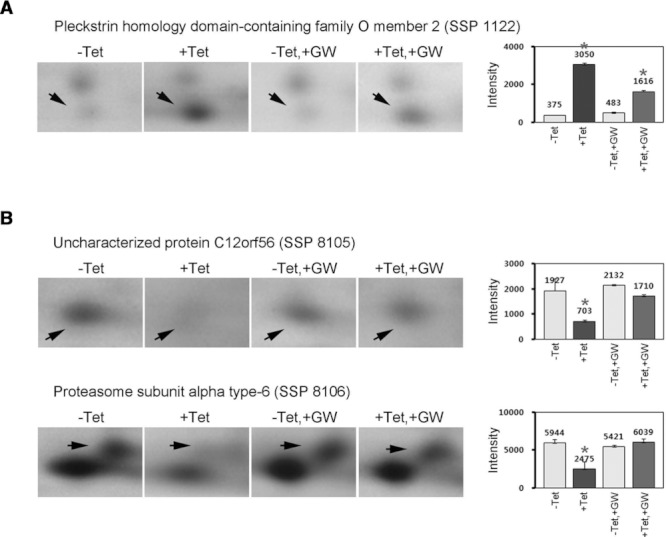
Quantitative analysis of GW441756 inhibitory effects on a potential TrkA-dependent protein spots. Three protein spots of SSP 1122, 8105, and 8106 were more than twofold upregulated (A) or downregulated (B) by TrkA, and it was significantly suppressed by GW441756.

In addition, we showed here that SSP 1122, 8105, and 8106 were potentially identified as pleckstrin homology domain-containing family O member 2, uncharacterized protein C12orf56 and proteosome subunit alpha type-6, respectively, by MALDI-TOF MS analysis. As shown in Fig. 5, these protein spots were more than twofold upregulated or downregulated by TrkA with a statistical significance (*p < 0.05), and it was significantly suppressed by GW441756 (Fig. 5A and B).

### 3.6 Characterization of novel TrkA-dependent targets associated with tyrosine phosphorylation signaling pathways

**Figure 6:**
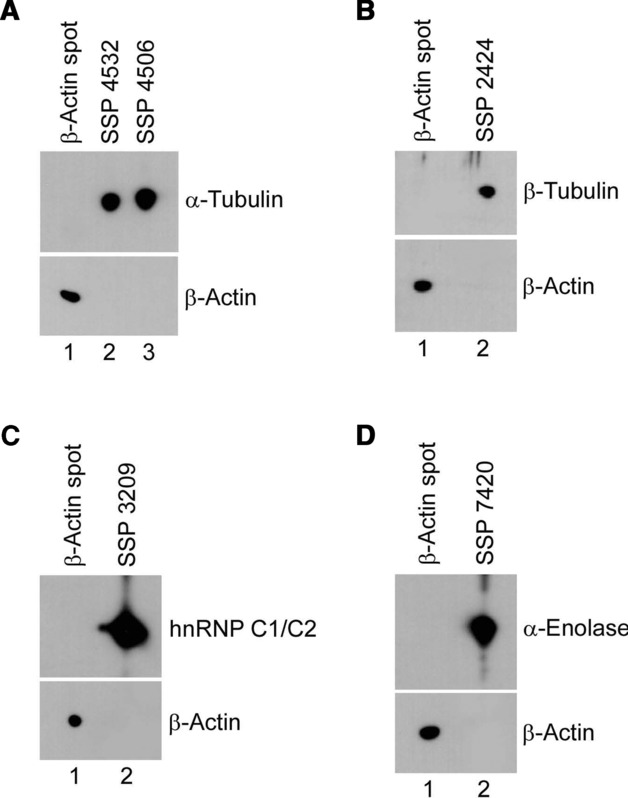
Verification of protein spots identified by MALDI-TOF MS and MALDI-TOF/TOF MS/MS analysis. Each protein spot and a piece of β-actin spot were excised from Commassie Blue stained gels and performed by 10% SDS-PAGE. The proteins were analyzed by Western blot using antibodies against α-tubulin (A), β-tubulin (B), hnRNP C1/C2 (C), and α-enolase (D). The NC membranes were reprobed by β-actin.

**Figure 7:**
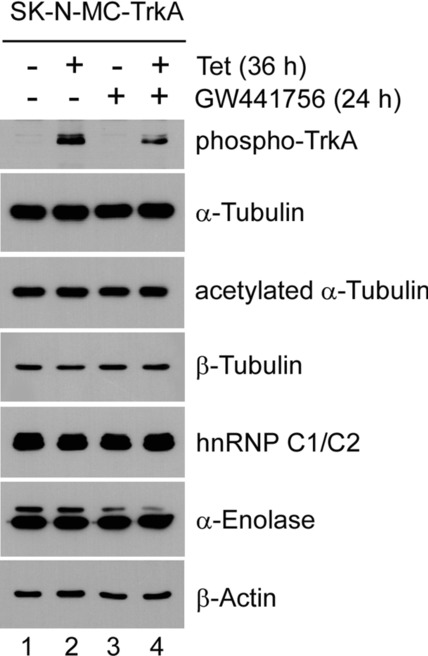
Investigation of TrkA and GW441756 effects on the identified proteins by 1DE/Western blot analysis. Protein samples (5 μg each) prepared for 2DE were separated by 10% SDS-PAGE and analyzed by Western blot using antibodies against phospho-TrkA, α-tubulin, acetylated α-tubulin, β-tubulin, hnRNP C1/C2, α-enolase, and β-actin.

**Figure 8:**
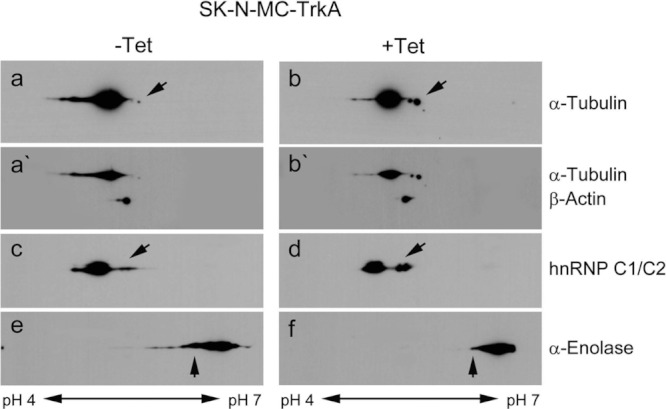
Characterization of TrkA effects on the identified proteins by 2DE/Western blot analysis. Protein samples (10 μg each) prepared for 2DE were performed by 1D isoelectric focusing using 7 cm IPG strip (pH 4–7), separated by 10% SDS-PAGE and transferred to NC membrane. Panels a and b were detected by Western blot analysis using α-tubulin antibody, and the blots were reprobed with α-tubulin and β-actin antibodies (panels a’ and b’). Panels c and d were detected with hnRNP C1/C2 antibody, and panels e and f were detected with α-enolase antibody. Each arrow indicates major area modified by TrkA.

**Figure 9:**
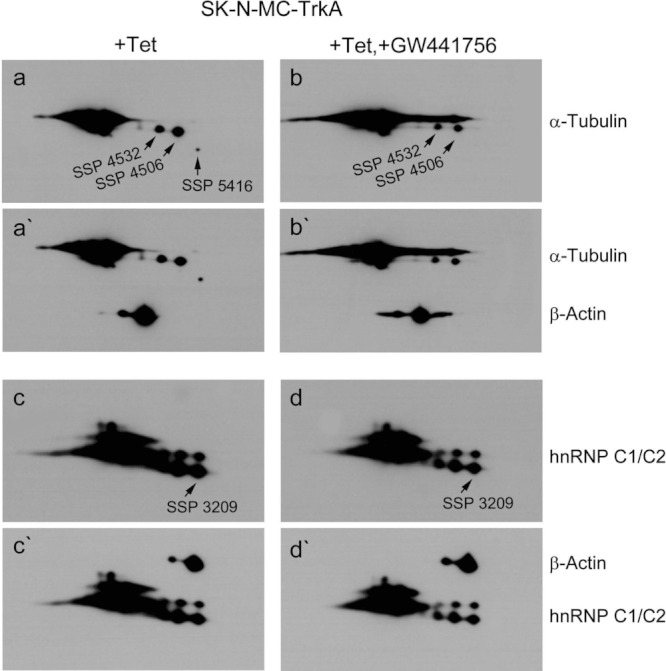
Confirmation of GW441756 effects on the identified protein spots by 2DE/Western blot analysis. Protein samples (50 μg each) were performed by 1D isoelectric focusing using 17 cm IPG strip (pH 4–7) and separated on 8.5–14% sucrose gradient polyacrylamide gels. The areas of α-tubulin and hnRNP C1/C2 protein spots were excised and transferred to NC membrane. Panels a and b were performed by Western blot analysis using α-tubulin antibody, and the blots were reprobed with α-tubulin and β-actin antibodies (panels a’ and b’). Panels c and d were detected with hnRNP C1/C2 antibody, and the blots were reprobed with hnRNP C1/C2 and β-actin antibodies (panels c’ and d’).

To probe the identification results, each protein spot and a piece of β-actin spot as a negative control were picked from Commassie Blue stained gels and analyzed by Western blot. As shown in Fig. 6, five protein spots were demonstrated as α-tubulin (6A, SSP 4532, and 4506), β-tubulin (6B, SSP 2424), hnRNP C1/C2 (6C, SSP 3209), and α-enolase (6D, SSP 7420). In Fig. 4A and B, we showed that these protein spots were either upregulated or downregulated by TrkA, and it was suppressed by GW441756. To probe this, the protein samples were performed by 10% SDS-PAGE followed by Western blot analysis. As expected, TrkA phosphorylaton at tyrosine-490 was significantly downregulated by GW441756 (Fig. 7, first panel). However, the identified major TrkA-dependent targets such as α-tubulin, β-tubulin, hnRNP C1/C2, and α-enolase were not affected by TrkA and GW441756, and acetylation of α-tubulin appeared to have no significant effect by TrkA (Fig. 7). Thus, the protein samples were performed by 2DE using 7 cm IPG strip (pH 4–7) and analyzed by Western blot. The results showed that certain modifications of α-tubulin and hnRNP C1/C2 were upregulated by TrkA, whereas α-enolase modification was downregulated by TrkA (Fig. 8, compare arrow areas between –Tet and +Tet panel). Finally, to prove that these modified protein spots are associated with TrkA-mediated tyrosine phosphorylation signaling pathways, the protein samples were performed by 2DE using 17 cm IPG strip (pH 4–7) and analyzed by Western blot. As shown in Fig. 9, certain modifications of α-tubulin (SSP 4532, 4506, 5416) and hnRNP C1/C2 (SSP 3209) by TrkA was significantly downregulated by GW441756. Taken together, our results suggest that these TrkA-dependent novel targets could play an important role in TrkA-mediated tyrosine phosphorylation signaling pathways via regulation of their posttranslational modifications.

## 4 Discussion

Paradoxically, TrkA plays an essential role in either cell survival or death depending on a cellular circumstance in an NGF-dependent or NGF-independent mechanism. Moreover, activation of TrkA via Y-490 phosphorylation is required for both TrkA-induced cell survival 33,34 and death 7,11,27. Until now, it is still unclear about the mechanisms why TrkA induces cell survival or death via Y-490 phosphorylation in an NGF-dependent or NGF-independent mechanism. To solve this puzzle, it seems to be very important to reveal the differences of signal transduction pathways between TrkA-induced cell survival and death. We have previously showed that TrkA overexpression induced apoptotic cellular processes including PARP and Bax cleavages in both neuronal SK-N-MC cells and nonneuronal U2OS cells, leading to the decrease of colony formation by cancer cells and induction of a significant cell death 23,28. Interestingly, TrkA-induced cell death was suppressed by the expression of caveolin-1 via downregulation of Y-490 phosphorylation 35, whereas it was enhanced by the expression of H2AX via upregulation of Y-490 phosphorylation 36, suggesting a critical role of Y-490 phosphorylation in TrkA-induced cell death. To better understand the mechanisms of TrkA-induced cell death, we identified novel targets associated with TrkA-mediated tyrosine phosphorylation signaling pathways using TrkA inhibitor GW441756 by proteomic analysis (Tables 1 and 2).

Microtubules, formed by the repeated connection of α- and β-tubulin heterodimer, are a major component of the cytoskeleton and play an important role in the maintenance of cell shape and integrity, mitosis, and intracellular transport 37. Isotypes of human α- and β-tubulin have been identified 38,39, and each isotype can undergo various posttranslational modifications such as acetylation, tyrosination, detyrosination, Δ2 modification, polyglutamylation, polyglycylation, palmitoylation, and phosphorylation 31,32. These modifications of tubulin affect microtubule function and organization 40. Moreover, it has been reported that α-tubulin is colocalized with phosphorylated TrkA, suggesting a

possibility of α-tubulin phosphorylation by TrkA 41. We showed here that certain modifications of α-tubulin were significantly upregulated by TrkA (Fig. 8, compare panels a and b), and it was significantly suppressed by GW441756 (Fig. 9, compare panels a and b), indicating that TrkA enzyme activity is required for the modifications of α-tubulin. These results suggest that TrkA-induced morphological change of neuroblastoma cells is largely associated with certain modifications of α- and β-tubulin.

hnRNP family members including hnRNP C1/C2 are nucleic acid-binding proteins that are mainly found in the 40S-ribonucleoprotein particle and have been implicated in the regulation of telomere and telomerase 42. hnRNP C1/C2 proteins are upregulated during staurosporine-induced apoptosis in HT22 cells 43 and can be specifically cleaved by interleukin 1beta-converting enzyme-like proteases during apoptosis 44. It has been known that hnRNP C1/C2 proteins are rapidly phosphorylated on several serine residues by hydrogen peroxide in human endothelial cells 45,46 and are involved in DNA damage repair signaling pathways via various protein modifications during ionizing radiation 47. In addition, the nuclear hnRNP C1/C2 proteins are translocated to the cytoplasm in a Rho-associated kinase (ROCK)-dependent manner during phorbol-12-myristate-13-acetate (PMA)-induced apoptosis 48. We showed here that certain modifications of hnRNP C1/C2 were significantly upregulated by TrkA (Fig. 8, compare panels c and d), and it was suppressed by GW441756 (Fig. 9, compare panels c and d). These results suggest that hnRNP C1/C2 could play a crucial role in the regulation of TrkA-induced cell death via, at least, tyrosine phosphorylation signaling pathway.

Peroxiredoxins are a family of antioxidant thioredoxin-dependent peroxidases and plays an important role in cellular protection against oxidative stress 49. Six different peroxiredoxin isozymes have been identified in different brain regions and different cell types, and the altered expression levels of these isozymes are associated with the various diseases including neurodegenerative disorders 50,51. Moreover, peroxiredoxins protect nonmalignant and malignant cells against radiation and chemotherapies 52, and peroxiredoxin-6-knockout mice had more significant hepatocellular injury compared with wild-type mice 53. In this report, we propose that peroxiredoxin-6 is a novel TrkA-dependent target associated with tyrosine phosphorylation signaling pathways (Fig. 4B), and TrkA-induced cell death is associated with the loss of cell protection function of peroxiredoxin-6.

Enolase is a glycolytic enzyme that catalyzes the conversion of 2-phosphoglycerate to phosphoenolpyruvate and plays an important role in glucose metabolism and Alzheimer's disease 54. In addition, it has ability to function as a plasminogen receptor on the cell surface, heat shock protein and transcriptional regulator 55. In mammals, three isoforms of the enzyme, α-, β-, and γ-enolase, have been identified with high sequence homology 56,57. Both upregulation and posttranslational modifications of α-enolase have been known to be involved in cancer invasion 57 and may have diagnostic value in pancreatic cancer 58,59. Notably, little has been studied about the interaction between TrkA and enolase. We showed here that α-enolase modification was significantly downregulated by TrkA (Fig. 8, compare panels e and f), suggesting an unknown function of α-enolase in TrkA-induced cell death.

Taken together, our results further support that abnormal high expression of TrkA selects cell death rather than survival in neuroblatoma cells via modulation of novel TrkA-dependent targets such as α- and β-tubulin, hnRNP C1/C2, peroxiredoxin-6, and α-enolase. Finally, we propose here that TrkA has been implicated in the regulation of glucose metabolism by downregulating of α-enolase modification.

## Abbreviations

ERK: extracellular signal-regulated protein kinase
hnRNP C1/C2: heterogeneous nuclear ribonucleoproteins C1/C2
IEF: isoelectric focusing
JNK: c-Jun N-terminal kinase
NGF: nerve growth factor
PLC-γ: phospholipase C-γ
PMF: peptide mass fingerprinting
Tet: tetracycline
TrkA: tropomyosin-related kinase A
